# Influences of Agrochemicals on Health and Ecology in Vietnamese Mango Cultivation

**DOI:** 10.1155/2021/6434309

**Published:** 2021-10-26

**Authors:** Kiet Hong Vo Tuan Truong, Nguyen Thi Pham, Thoa Thi Kim Nguyen

**Affiliations:** ^1^Socio-Economics and Policy Studies, Mekong Delta Development Research Institute, Can Tho University, Can Tho, Vietnam; ^2^Agricultural and Food Policy Studies, Institute of Tropical Agriculture and Food Security, Putra University, Seri Kembangan, Malaysia; ^3^SEARCA's Scholar, Laguna, Los Banos, Philippines; ^4^Department of Agricultural Economics, School of Economics, Can Tho University, Can Tho, Vietnam

## Abstract

The study aims to identify risks of agrochemicals that impact farmworkers, consumers, and ecology in Vietnamese mango cultivation to enhance safety and friendly production. The study finds out the total numbers of root fertilizers (N-P-K) of the noncooperative and cooperative farmers are similar, approximately 1,400 kg/ha/year higher than those in other countries. Excessive fertilizer usage is a potential threat to soil, water, and air pollution. In addition, the findings indicate that the ecology component is undergoing the most negative impact from excessive agrochemical use in mango farming. The vast majority of agrochemicals in mango cultivation are fungicide and paclobutrazol over 90% of the total number of agrochemicals used in both noncooperative and cooperative farmer groups among the three seasons. Total field EIQ of the cooperative grower category is less than that of the noncooperative grower category. These results show that mango cultivation should consider rejecting the banned active ingredients of glyphosate, paraquat, and carbendazim as well as reducing fungicide and paclobutrazol usage and encouraging cooperative participation to safeguard the environment and human health. Moreover, science information needs to be closely linked and fed back to policy development to boost the management of the awareness of the ecological risks for farmers associated with reducing agrochemical use in mango cultivation.

## 1. Introduction

Nowadays, agriculture production relies on increasingly agrochemical and chemical fertilizers to increase productivity [1]. Farmers overuse various active ingredients to control weeds, pests, and diseases in crops [2]. Although the main purpose focuses on harmful organisms, it also influences the nontarget system. Moreover, most of the growers are unaware of the potential toxicities of synthetic agrochemicals as well as the lack of information about the level of poisoning, hazards, and safety measures [3]. This has led to a substantial impact on not only the ecosystem (biodiversity loss, soil, and water resources pollution) but also human health from general poisoning to carcinogenic effects [4–7].

Mango is one of the most popular tropical fruits, known as the king of fruits, nutritionally rich fruits with unique flavour, fragrance, and taste. Vietnam is a tropical country with long latitude, sun, heavy rain, diverse ecology, and clear zoning. The most prominent advantage is tropical fruits such as durian, rambutan, mangosteen, mango, jackfruit, guava, and dragon fruit. Mango is the second most popular fruit in Vietnam (after banana) and is grown across many provinces. Mango is one of the most prominent tropical fruits in Vietnam, and its production generates a large amount of income for growers and stakeholders in the value chain. The production area of mango rose sharply between 2015 (83.7 thousand hectares) and 2019 (104 thousand hectares). The Mekong Delta (MD) is the key region for tropical fruit production in Vietnam. Due to its ideal natural conditions and effective farming techniques, the MD has the largest share of mango production in Vietnam. The region also has the highest overall productivity level in Vietnam and plays a key role in the government's flowering manipulation policy. In 2019, it was the largest mango production region in the country, with 48,200 ha (46.3% of the national total) and 511,800 tons (62.8% of the national total). Within the region, the largest mango areas are in Dong Thap, An Giang, Vinh Long, Hau Giang, Dong Nai, and Tien Giang [8]. Farming mango season in Vietnam is produced actively year-round by implementing the flowering stimulation technique. Hence, the quantity of agrochemicals used in crops is more frequent.

There are different measurements of the environmental risk of agrochemical use [9]. However, assessments of pesticide applications still use EIQ for scientific and policy purposes [10–13]. The use of EIQ has been debated in [14–16]. 

In the recent literature, various studies have determined a nexus of human health damage and air pollution [17, 18]. Similarly, Elahi et al. [19] evaluated a nexus between the use of agrochemicals and health damage and found that the rampant use of agrochemicals in crops damaged human health. Although previous studies have focused on the use of agrochemicals and human health damage, limited studies have focused on the influences of agrochemicals on health and ecology for mango production in Vietnamese. Therefore, this study determines the current status of fertilizer and pesticide (herbicide, insecticide, and fungicides) use in mango crops to evaluate the potential hazards of synthesis chemical overuse on human health and the environment. This is a contribution to the sustainability of mango production for the healthy type of producers, consumers, and ecology as well as suggesting evidence-based policy decisions associated with the future management of agrochemical use.

## 2. Methodology

### 2.1. Conceptual Underpinning of EIQ

The environmental impact quotient (EIQ) model was developed by Kovach et al. [20] at Cornell University to measure the impacts of different crop pests and disease managements on health and the ecology. The method addresses a majority of the environmental concerns that are encountered in agricultural systems including farmworkers, consumers, wildlife, health, and safety.

Table 1 provides information on eleven variables used to establish the EIQ formula. These parameters are divided into three levels (scores 1, 3, and 5) to identify its effects, with 1 representing the lowest, 3 intermediate, and 5 the highest. Besides, the evaluation of the potential risks of agrochemical toxicity is considered by LD50 (dose at 50% of the treatment category dies within a given period), LC50 (concentration at 50% of the treatment category dies within a given period), and the potential exposure comprising half-life, runoff, or leaching potential [22]. After that, these scores are aggregated to compute the final composite EIQ score for each pesticide's active ingredient.

Information from Table 2 presents how to establish the EIQ formula of three components (farmworker, consumer, and ecology) based on the effects of agrochemicals on the environment into eight categories. In detail, the farmworker category includes potential effects to applicators and field workers; the consumer category comprises the potential effects of residues on the consumer and groundwater contamination. Groundwater effects are included in the consumer component because it is more of a human health issue (drinking contaminated water) than a wildlife issue. The ecological category encompasses the potential effects on aquatic organisms, bees, birds, and beneficial arthropods. Next, the total of farmworkers, consumers, and ecology is an average value of three EIQ components (1). To calculate the field EIQ for an individual agrochemical, it is the total of the EIQ of the individual active ingredients to multiple percentages of each active ingredient and the application rate of the formulation (2). If values of the field EIQ are higher and higher, it will lead to mango cultivation greater risk.

Calculation of total field EIQ helps producer and farm manager have overall picture of agrochemicals use in production process and identify the negative impacts of these pesticides on health and ecology. This helps them have necessary adjustments in pesticide use for pests and disease management more environmentally friendly [20]. In this paper, all calculations of the EIQ values were done using Cornell University's online EIQ calculator in May 2020 (Table 3).

### 2.2. Data Sources

**Figure 1:**
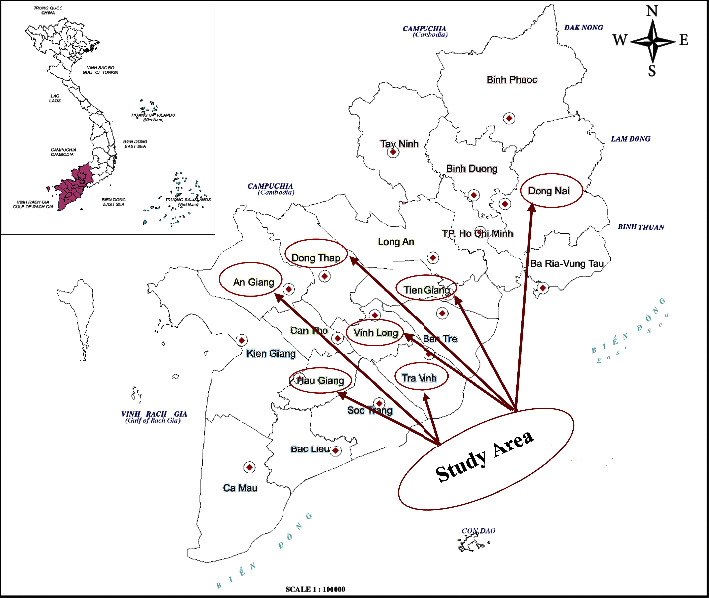
Study areas in southern Vietnam. Source: design by author.

Our data cover seven of the major mango farming provinces in southern Vietnam such as An Giang, Dong Thap, Tien Giang, Vinh Long, Tra Vinh, Hau Giang, and Dong Nai (Figure 1). The data collection was carried out in multistage. First, we discussed with agricultural extension workers at the province and district levels to choose mango villages. Second, we had 14 discussion groups (4–6 people per group) in fourteen villages to determine the essential factors of mango cultivation before designing the questionnaire. Third, the study conducted a trial survey with 84 sampling observations (12 observations in each province). Finally, a simple random technique was used to select 1,886 sampling observations for computing the EIQ of human health and environmental impacts, in which 818 sampling observations were of the cooperative farmer groups (285, 262, and 271 for seasons 1, 2, and 3, respectively) and 1,068 sampling observations of the noncooperative farmer group (361, 415, and 292 for seasons 1, 2, and 3, respectively).

## 3. Results and Discussion

### 3.1. The Situation of Chemical Fertilizer Use in Mango Cultivation in Southern Vietnam

Although there are at least fourteen elements or nutrients that are required for plant growth, the three key nutrients for mango production are nitrogen (N), potassium (K), phosphorous (P) [23]. Understanding the interactions of these 3 nutrients is the key to good productivity and fruit quality in mangoes. This is the most important element for the mango crop, due to its strong influence on vegetative growth, flowering, yield, and fruit quality. Its concentration in plant tissues influences considerably the concentrations and effects of other nutrients [24]. The positive effect of N fertilization on mango yield was clearly indicated in Florida by [25]. They also found a good correlation between treatment and leaf concentration of N and K and observed that those leaf levels tended to decrease after a heavy crop [26].

Data from the Southern Vietnam survey (Table 4) show that the total number of used root fertilizers (N-P-K) of the noncooperative and cooperative farmers is similar, approximately 1,400 kg/ha/year (Table 4). However, there is a disparity in each season. More specifically, the used root fertilizer (N-P-K) of the cooperative farmers is 130.4 kg less than that of the noncooperative farmers in season 1. Similarly, this figure in season 2 is 123.6 kg. On the other hand, the number of cooperative farmers in season 3 is 234.3 kg more than the noncooperative farmers. The finding indicates that root fertilizer (N-P-K) use of mango growers in southern Vietnam is relatively high compared to other countries. In more detail, the cooperative grower category is 537.3, 480.5, and 362.6 kg/ha in seasons 1, 2, and 3, respectively, and the noncooperative grower category is 667.7, 604.1, and 128.3 kg/ha in seasons, 1, 2, and 3, respectively. The figure is 100.4 in Turkey, 665.5 in the Netherlands, 624.8 in Egypt, 373.2 in Japan, 301.5 in China, 287.5 in Britain, 205.4 in Germany, 180.1 in France, 160.8 in the USA, 126.4 in Italy, 121.4 in India, 115.4 in Greece, and 106.9 in Indonesia kg/ha, respectively [4]. In addition, the root fertilizer (N, P, K) use in seasons 1 and 2 of the noncooperative farmer group was higher than that of [27]; however, these findings of the cooperative farmer group are lower than [27] in those in the three cropping seasons.

Applying the N fertilizer in mango cultivation also needs to note that if applied excessively, it can have harmful impacts such as reduction of yellow skin percentage in mature fruits, increasing the severity of anthracnose [28]. Meanwhile, potassium is an essential factor in the progress of thick epidermal cell walls, maintaining the ionic strength of the cytoplasm, and water uptake and water loss through the stomata. This contributes to boosting the resistance of trees to pests and diseases [29, 30]. In addition, the result of [31] stated that the increase in potassium fertilizer use has been a positive relationship with fruit weight, flavour, colour, and shelf life. A report of [32] mentioned that the combination of phosphorous with N and K has contributed to increased mango yield. Particularly, phosphorus can promote the development of roots, branches, seeds, and fruits, and the absorption of water and nutrients.

In mango farming, mango gardeners usually apply the liquid fertilizer (N-P-K) to spray on mango leaves for flowering stimulation (Table 4). The liquid fertilizer consists of a large number of microelements (Mn, Zn, Cu, Mo, S, and B) compared to the root fertilizer. Overall, the liquid fertilizer (N-P-K) usage of noncooperative and cooperative farmers is similar, in which the two main components are nitrogen and potassium. According to [30], potassium nitrate has played an important role in flowering stimulation for mango cultivation and has increased flower induction, fruit set, and fruit retention.

In summary, the application of fertilizer in mango production needs strict control because it has not only an influence on humans (farmworkers, consumers) but also an impact on ecology (soil, water, air, and terrestrial and aquatic ecosystems). Nitrogenous fertilizer is considered one of the most important inputs in agricultural production. Its overuse causes air pollution by nitrogen oxide (NO, N_2_O, NO_2_) emissions. The nitrogenous fertilizer used in mango cultivation only absorbs a part of the soil, the rest of the nitrogenous fertilizer is lost by evaporation and reacts with organic compounds in the clay soil, and the remaining interferes with surface and groundwater [33].

### 3.2. Human Health and Environment Impacts of Mango Cultivation

In general, there are several active ingredients (herbicide, insecticide, and fungicide) that are applied in mango production, in which nineteen active ingredients (Table5) are the most popular elements. According to the classification of [34], the total applied agroinputs of mango farming in southern Vietnam, none are classified in category Ia (extremely hazardous). Category Ib (highly hazardous) is the abamectin, category II (moderately hazardous) includes the paclobutrazol, paraquat, 2,4-D, cypermethrin, chlorpyrifos, emamectin, imidacloprid, permethrin, ziram, difenoconazole, and tebuconazole. Glyphosate and metalaxyl are the list of category III (slightly hazardous), and mancozeb, propineb, carbendazim, azoxystrobin, and trifloxystrobin are in category U (unlikely to present an acute hazard when in regular use).

The results of Table 5 indicated that paraquat, permethrin, and carbendazim are the list of banned active ingredients. Both paraquat and carbendazim are acute toxicity characteristics that cause health problems soon after exposure. Particularly, paraquat is highly toxic to animals and humans and is blamed for causing severe, acute, and long-term health problems and is banned in most countries. Besides, the active ingredients of paclobutrazol, glyphosate, 2,4-D, abamectin, imidacloprid, mancozeb, and ziram are the list of controlled elements in farming. For example, abamectin and ziram are acute toxicity “fatal if inhaled.” Active ingredients of 2,4-D mancozeb are listed as “endocrine disruptors or potential endocrine disruptors” in EU regulation. Chlorpyrifos and imidacloprid are potentially causing bee colony collapse disorders which are acutely and highly toxic to honey bees. Glyphosate is probably likely carcinogenic. Hence, mango cultivation needs to encourage farmers to use lower hazardous agrochemicals as well as increasing awareness of less hazardous chemical use. It helps in safeguarding the environment and human health.

The findings from Table 6 show that the total field EIQ in the noncooperative and cooperative farmer groups are 1,303.23 and 1,083.93 kg/ha. The number of the noncooperative farmer group is 244.67 kg/ha higher than the study result by [27], and this figure of the cooperative farmer group is similar to the finding in [27]. Permethrin is the lowest with 0.07 and 0.05 in the noncooperative and cooperative farmer groups, whereas the highest is for the paclobutrazol 640.43 in the noncooperative grower group and 534.53 in the cooperative grower group.

According to [36], EIQ classification values for all agrochemicals used in mango production showed that 30%, 25%, and 45% of those agrochemicals have been rated as low (EIQ = 0 to 20), moderate (EIQ = 21 to 40), and high (EIQ ≥41), respectively. The findings of the study (Table 7) show that the active ingredients that have field EIQ of more than 41 are paclobutrazol, mancozeb, and propineb in the noncooperative and cooperative farmer groups. Generally, the results indicate that the ecology component of the EIQ is higher compared with farmworker and consumer components in the noncooperative and cooperative farmer, but there is a significant difference in the field EIQ between the two categories of farmers.

The field EIQ values of the farmworkers, consumers, and ecology components in the cooperative farmers are 616.08, 319.51, and 1,778.86, respectively (Table 7). These figures are lower than compared with those of the noncooperative farmers among the three seasons. It means that the farmworkers, consumers, and ecology components of the cooperative farmers are 39.29, 81.03, and 244.30, respectively, less than those of the noncooperative farmers. Particularly, the field EIQ value of ecology components is the highest in the three EIQ components in the noncooperative and cooperative farmers. In the cooperative farmer group, the ecology factor is 2.9, 5.6 times greater than the farmworker and consumer factor. In the noncooperative farmer group, these numbers are 3.1 and 5.1 times. The result implies that the ecology is undergoing the most negative impact from excessive agrochemical use in mango production. In general, the total field EIQ of the cooperative grower category is 121.54, less than that of the noncooperative grower category. In particular, the total field EIQ values in the noncooperative and cooperative farmer groups are 1,026.26 and 904.72 kg/ha, and these figures are 279.2 and 157.66 kg/ha greater than those in the previous research [27].

The findings of season 3 (Table 8) are similar to those of season 2 (Table 7). The result shows that the field EIQ values of farmworkers, consumers, ecology components and total EIQ values in the noncooperative farmers are 1.17, 1.38, 1.26, and 1.26 times greater than those of the cooperative farmers, implying that the cooperative farmer production is more positive than the noncooperative production. Specifically, the total field EIQ in the noncooperative and cooperative grower categories are 813.89 and 648.41 kg/ha, and these numbers are 221.55 and 56.07 kg/ha higher than the finding in [27].

Among the agrochemicals, fungicide has the highest EIQ percentage compared to paclobutrazol, herbicide, and pesticides. Specifically, the fungicide EIQ percentage of the cooperative grower group is 42.9%, 52.5%, and 69.6% in seasons 1, 2, and 3, respectively, and in the noncooperative grower group, it is 45.3%, 61.3%, and 75.4% in seasons 1, 2, and 3, respectively. Next is paclobutrazol that is an active ingredient of mango growth control to stimulate flowering. Paclobutrazol EIQ rate of the cooperative farmers is 49.3% in season 1, 42.0% in season 2, and 23.2% in season 3, and the figure of the noncooperative farmers is 49.1% in season 1, 31.2% in season 2, and 16.6% in season 3. The result of the study indicates that the vast majority of agrochemicals in mango cultivation is fungicide and paclobutrazol over 90% of the total amount of agrochemical used in the noncooperative and cooperative farmer groups among the three seasons.

In herbicides, glyphosate is used the most popular to control weeds in both noncooperative and cooperative farmer groups among the three seasons, followed by paraquat. The glyphosate may reach groundwater, surface water, and other nontarget sites through processes of runoff, spray drift, and root uptake. The herbicide glyphosate raises the susceptibility of plants to diseases and impacts the health and nutrition of crops [37]. In fungicides, mancozeb is the most active ingredient in all of the fumigants. The fungicide mancozeb remarkably impacts soil microflora, nitrification, ammonification, soil microbial biomass, carbon mineralization, and soil enzymes that may have a negative influence on nutrient uptake and plant growth.

## 4. Conclusions and Policy Implications

The study contributes to the interpretation of threats of fertilizers and pesticides to health and ecology in mango farming in Vietnam by the EIQ model. The findings show that the cooperative farmer group uses the N-P-K fertilizers more than the noncooperative farmer group. The total field EIQ of the cooperative grower category is less than that of the noncooperative grower category. The ecology EIQ component is higher than the farm worker and consumer EIQ components in the noncooperative and cooperative farmer group in three seasons. The ecology is undergoing the most negative impact from excessive agrochemical use in mango farming. The vast majority of agrochemicals in mango cultivation is fungicide and paclobutrazol over 90% of the total number of agrochemical used in the noncooperative and cooperative farmer groups among the three seasons.

To reduce agrochemical use in mango production, farmers should focus on reducing fungicide and paclobutrazol usage. To safeguard the environment and human health, mango cultivation in southern Vietnam should consider rejecting glyphosate, paraquat, and carbendazim. These active ingredients are acute toxicity characterized with high toxicity to animals and humans that caused health problems soon after exposure to UTZ classification in 2015. Moreover, the N-P-K used in mango cultivation needs to be considered carefully because overuse of the fertilizer may lead to negative effects on mango progression and soil, water, air, and terrestrial and aquatic ecosystems.

Agrochemicals are widely used in mango cultivation to ensure crop quality and production. However, the use of such agrochemicals may cause adverse effects to the farmworkers, consumers, and ecology. It has long-term effects on human health. Agrochemical pollution is not easily measured, so the potential environmental risks do not evoke an immediate response, as they can occur in several years. Therefore, science information needs to be closely linked and fed back to policy development to boost the management of the awareness of the ecological risks for farmers associated with reducing agrochemical use in mango cultivation.

## Figures and Tables

**Table 1:** Definition of symbols and ratings for each toxicity category.

Variables	Symbol	Score 1	Score 3	Score 5
(1) Long-term health effects (chronic)	C	Little–none	Possible	Definite
(2) Dermal toxicity (rat LD50)	DT	>2000 mg/kg	200–2000 mg/kg	0–200 mg/kg
(3) Bird toxicity (8-day LC50)	D	>1000 ppm	100–1000 ppm	1–100 ppm
(4) Bee toxicity	Z	Nontoxic	Moderately toxic	Highly toxic
(5) Beneficial arthropod toxicity	B	Low impact	Moderate	Severe impact
(6) Fish toxicity (96 h LC50)	F	>10 ppm	1–10 ppm	<1 ppm
(7) Plant surface half-life	S	1-2 weeks	2–4 weeks	>4 weeks
(8) Soil residue half-life (TI/2)	P	<30 days	30–100 days	>100 days
(9) Mode of action	SY	Nonsystem	Systemic	
(10) Leaching potential	L	Small	Medium	Large
(11) Surface runoff potential	R	Small	Medium	Large

Source: [20, 21].

**Table 2:** environmental components of the EIQ equation.

EIQ equation component	Equation
Farmworker (applicator + harvester)	c^*∗*^((dt^*∗*^5) + (dt^*∗*^p))
Consumer (exposure + groundwater effects)	(c^*∗*^(*s* + *p*)/2^*∗*^sy) + (L)
Ecology (fish, birds, bees, other beneficial insects)	(f^*∗*^r) + (d^*∗*^(*s* + *p*)/2^*∗*^3) + (z^*∗*^p^*∗*^3) + (b^*∗*^p^*∗*^5)
Total EIQ = farmworker + consumer + ecology	
{[c^*∗*^(dt^*∗*^5) + (dt^*∗*^p)] + [(c^*∗*^(*s* + *p*)/2^*∗*^sy) + (L)+[(f^*∗*^r)+(d^*∗*^(*s* + *p*)/2^*∗*^3) + (z^*∗*^p^*∗*^3) + (b^*∗*^p^*∗*^5)]}/3 (1)	
Field use EIQ = EIQ ^*∗*^ % active ingredient ^*∗*^ rate/ha (2)	

Source: [20].

**Table 3:** The theatrical values of EIQ by Cornell University.

Active ingredient	EIQ component value			EIQ value
	Farmworker	Consumer	Ecology	
Paclobutrazol	21.30	6.55	51.45	26.43

*Herbicide*				
Glyphosate	8.00	3.00	35.00	15.33
Paraquat	31.95	6.33	35.92	24.73
2,4-D Dimethylamine	24.00	7.00	31.00	20.67

*Insecticide*				
Cypermethrin	13.80	5.90	89.35	36.35
Chlorpyrifos	6.00	2.00	72.55	26.85
Emamectin benzoate	9.00	4.00	65.85	26.28
Abamectin	13.80	3.90	86.35	34.68
Imidacloprid	6.90	10.35	92.88	36.71
Permethrin	12.00	5.00	71.00	29.33

*Fungicide*				
Mancozeb	20.25	8.13	48.79	25.72
Propiconazole	12.00	19.00	63.90	31.63
Ziram	24.00	9.00	42.45	25.15
Carbendazim	25.00	40.50	86.00	50.50
Difenoconazole	15.00	23.50	86.00	41.50
Tebuconazole	20.00	31.00	70.00	40.33
Azoxystrobin	8.10	6.05	66.62	26.92
Metalaxyl	8.10	12.15	36.95	19.07
Trifloxystrobin	12.15	10.23	66.95	29.78

Source: [20] and EIQ values updated in 2020.

**Table 4:** The number of chemical fertilizers in mango cultivation in southern Vietnam.

Items	Season 1			Season 2			Season 3		
	Non-coop. (*n* = 361)	Coop. (*n* = 285)	*t* -test	Non-coop. (*n* = 415)	Coop. (*n* = 262)	*t*-test	Non-coop. (*n* = 292)	Coop. (*n* = 271)	*t* -test
Root fertilizer									
N (kg)	294.9	215.1	^*∗∗*^	251.0	189.0	*ns*	49.2	146.7	^*∗∗*^
P (kg)	201.5	159.0	*ns*	190.0	130.0	*ns*	43.4	116.8	^*∗*^
K (kg)	171.3	163.2	*ns*	163.1	161.5	*ns*	35.7	99.1	^*∗∗*^
Microelements (gr)	27.9	35.7	*ns*	5.7	82.9	*ns*	0.5	40.9	*ns*
Leaf fertilizer (liquid) for flowering stimulation									
N (kg)	8.0	7.5	*ns*	8.1	6.0	^*∗∗*^	6.4	4.6	^*∗∗*^
P (kg)	2.8	1.0	^*∗∗*^	2.6	0.7	^*∗∗∗*^	2.5	0.5	^*∗∗∗*^
K (kg)	13.1	13.1	*ns*	12.3	10.4	*ns*	8.5	8.7	*ns*
Microelements (gr)	58.4	37.5	*ns*	124.6	52.8	^*∗∗∗*^	110.5	61.2	*ns*

Source: Field Survey Data, 2018. Unit: kg/ha. ^*∗*^ Significant at the 10% level, ^*∗∗*^ significant at the 5% level, ^*∗∗∗*^ significant at the 1% level, and ns: nonsignificant.

**Table 5:** The classification of active ingredients by WHO and UTZ.

Active ingredient	WHO class	UTZ class	
		Ban	Control
Paclobutrazol	II		
Herbicide			
Glyphosate	III		x
Paraquat	II	x	
2,4-D Dimethylamine	II		x
Insecticide			
Cypermethrin	II		
Chlorpyrifos	II		x
Emamectin	II		
Abamectin	Ib		x
Imidacloprid	II		x
Permethrin	II	x	
Fungicide			
Mancozeb	U		x
Propineb	U		
Ziram	II		x
Carbendazim^*∗∗*^	U	x	
Difenoconazole	II		
Tebuconazole	II		
Azoxystrobin	U		
Metalaxyl	III		
Trifloxystrobin	U		

Source: Field Survey Data, 2018 [34,35]. ^*∗∗*^ Pesticides meeting indicators of the list of banned pesticides but too challenging to be replaced by UTZ 2015.

**Table 6:** The practical values of health and environment impacts (EIQ) in season 1.

Active ingredient	EIQ component value						EIQ average value	
	Farmworker		Consumer		Ecology			
	Non-coop.	Coop.	Non-coop.	Coop.	Non-coop.	Coop.	Non-coop.	Coop.
(1) Paclobutrazol	516.12	430.78	158.71	132.47	1,246.69	1,040.54	640.43	534.53
(2) Herbicide	13.35	7.65	3.73	2.14	27.37	18.16	14.81	9.31
Glyphosate	3.55	2.88	1.33	1.08	15.53	12.60	6.80	5.52
Paraquat	4.85	3.61	0.96	0.72	5.45	4.06	3.75	2.79
2,4-D	4.95	1.16	1.44	0.34	6.39	1.50	4.26	1.00

(3) Insecticide	17.98	12.84	8.43	6.14	145.54	107.9	57.31	42.29
Cypermethrin	8.88	5.35	3.80	2.29	57.50	34.63	23.39	14.09
Chlorpyrifos	3.38	2.88	1.13	0.96	40.84	34.85	15.11	12.90
Emamectin	3.08	2.72	1.37	1.21	22.51	19.89	8.98	7.94
Abamectin	1.47	0.93	0.41	0.26	9.18	5.79	3.69	2.33
Imidacloprid	1.14	0.94	1.71	1.41	15.34	12.61	6.06	4.98
Permethrin	0.03	0.02	0.01	0.01	0.17	0.13	0.07	0.05

(4) Fungicide	366.99	308.57	270.34	228.35	1,134.89	956.61	590.68	497.80
Mancozeb	190.19	144.32	76.36	57.94	458.24	347.71	241.56	183.30
Propiconazole	78.89	75.59	124.91	119.68	420.08	402.50	207.93	199.23
Ziram	68.85	72.81	25.82	27.30	121.77	128.78	72.15	76.30
Carbendazim	11.81	3.61	19.14	5.85	40.63	12.41	23.86	7.29
Difenoconazole	7.81	5.92	12.24	9.27	44.80	33.93	21.62	16.37
Tebuconazole	5.41	4.05	8.39	6.27	18.94	14.16	10.91	8.16
Azoxystrobin	3.26	1.81	2.43	1.35	26.80	14.88	10.83	6.01
Metalaxyl	0.63	0.43	0.95	0.65	2.89	1.98	1.49	1.02
Trifloxystrobin	0.14	0.05	0.11	0.04	0.74	0.26	0.33	0.12
Field use EIQ	914.42	759.83	441.21	369.09	2,554.48	2,123.21	1,303.23	1,083.93

Source: Field Survey Data, 2018; unit: kg/ha.

**Table 7:** The practical values of health and environment impacts (EIQ) in season 2.

Active ingredient	EIQ component value						EIQ average value	
	Farmworker		Consumer		Ecology			
	Non-coop.	Coop.	Non-coop.	Coop.	Non-coop.	Coop.	Non-coop.	Coop.
(1) Paclobutrazol	257.93	306.44	79.32	94.23	623.03	740.21	320.05	380.25
(2) Herbicide	9.51	8.11	2.84	2.70	24.89	28.03	12.42	12.94
Glyphosate	4.25	5.78	1.60	2.17	18.61	25.29	8.15	11.08
Paraquat	3.05	1.57	0.60	0.31	3.42	1.76	2.36	1.21
2,4-D	2.21	0.76	0.65	0.22	2.86	0.98	1.90	0.65

(3) Insecticide	19.52	11.38	10.13	5.46	165.43	94.14	65.02	36.99
Cypermethrin	9.04	4.41	3.86	1.88	58.51	28.54	23.81	11.61
Chlorpyrifos	3.89	2.21	1.30	0.74	47.03	26.68	17.40	9.87
Emamectin	2.79	2.66	1.24	1.18	20.38	19.48	8.13	7.77
Abamectin	1.62	1.24	0.46	0.35	10.17	7.76	4.08	3.12
Imidacloprid	2.18	0.87	3.27	1.30	29.34	11.68	11.60	4.62
Permethrin	0.00	0.00	0.00	0.00	0.00	0.00	0.00	0.00

(4) Fungicide	368.41	290.14	308.26	217.13	1,209.80	916.48	628.77	474.54
Mancozeb	158.53	154.47	63.65	62.02	381.97	372.17	201.36	196.19
Propiconazole	101.12	72.85	160.10	115.34	538.44	387.92	266.53	192.02
Ziram	70.96	48.23	26.61	18.09	125.50	85.31	74.36	50.55
Carbendazim	20.60	3.90	33.38	6.32	70.88	13.42	41.62	7.88
Difenoconazole	8.61	5.47	13.49	8.57	49.38	31.35	23.83	15.13
Tebuconazole	5.12	2.94	7.94	4.56	17.93	10.31	10.33	5.94
Azoxystrobin	2.66	1.52	1.99	1.13	21.89	12.47	8.85	5.04
Metalaxyl	0.64	0.69	0.96	1.03	2.93	3.14	1.51	1.62
Trifloxystrobin	0.16	0.07	0.13	0.06	0.87	0.38	0.39	0.17
Field use EIQ	655.37	616.08	400.54	319.51	2,023.16	1,778.86	1,026.26	904.72

Source: Field Survey Data, 2018; unit: kg/ha.

**Table 8:** The practical values of health and environment impacts (EIQ) in season 3.

Active ingredient	EIQ component value						EIQ average value	
	Farmworker		Consumer		Ecology			
	Non-coop.	Coop.	Non-coop.	Coop.	Non-coop.	Coop.	Non-coop.	Coop.
(1) Paclobutrazol	109.05	121.48	33.54	37.36	263.42	293.44	135.32	150.74
(2) Herbicide	7.55	7.12	2.24	2.30	18.58	22.81	9.46	10.74
Glyphosate	2.99	4.50	1.12	1.69	13.06	19.69	5.72	8.62
Paraquat	2.29	1.57	0.45	0.31	2.57	1.76	1.77	1.21
2,4-D	2.28	1.05	0.67	0.31	2.95	1.36	1.96	0.90

(3) Insecticide	16.32	11.15	8.60	5.48	141.39	89.40	55.44	35.34
Cypermethrin	6.34	4.59	2.71	1.96	41.04	29.70	16.70	12.08
Chlorpyrifos	3.51	1.53	1.17	0.51	42.46	18.53	15.71	6.86
Emamectin	2.92	3.01	1.30	1.34	21.39	22.01	8.54	8.79
Abamectin	1.56	1.12	0.44	0.32	9.77	7.02	3.92	2.82
Imidacloprid	1.98	0.90	2.97	1.35	26.69	12.13	10.55	4.79
Permethrin	0.01	0.00	0.00	0.00	0.04	0.00	0.02	0.00

(4) Fungicide	361.70	282.10	300.23	205.32	1,179.26	867.48	613.67	451.59
Mancozeb	168.23	143.80	67.54	57.73	405.32	346.46	213.67	182.64
Propiconazole	93.85	67.33	148.59	106.60	499.74	358.52	247.37	177.47
Ziram	59.57	58.15	22.34	21.81	105.36	102.85	62.42	60.94
Carbendazim	24.44	4.11	39.59	6.66	84.06	14.14	49.36	8.31
Difenoconazole	7.63	3.93	11.96	6.15	43.76	22.51	21.12	10.86
Tebuconazole	4.77	3.16	7.39	4.90	16.68	11.06	9.61	6.37
Azoxystrobin	2.63	1.21	1.96	0.90	21.60	9.96	8.73	4.02
Metalaxyl	0.56	0.32	0.84	0.49	2.54	1.48	1.31	0.76
Trifloxystrobin	0.04	0.09	0.03	0.07	0.19	0.48	0.09	0.22
Field use EIQ	494.63	421.85	344.60	250.46	1,602.65	1,273.12	813.89	648.41

Source: Field Survey Data, 2018; unit: kg/ha.

## Data Availability

The data that support the findings of this study are available from the Technology and Science Program for Sustainable Development in Mekong Delta Region but restrictions apply to the availability of these data, which were used under license for the current study and so are not publicly available. Data are, however, available from the authors upon reasonable request and with the permission of the Technology and Science Program for Sustainable Development in Mekong Delta Region.
